# Whole-Body Vibration Exercise in Different Postures on Handgrip Strength in Healthy Women: A Cross-Over Study

**DOI:** 10.3389/fphys.2020.469499

**Published:** 2021-01-12

**Authors:** Luciana M. M. Santos, Ana Carolina C. Oliveira, Sueli F. Fonseca, Angélica F. Silva, Joyce N. V. Santos, Ana Lúcia C. Souza, Jousielle M. Santos, Vanessa G. C. Ribeiro, Arthur N. Arrieiro, Ana Caroline N. Prates, Luana A. Soares, Pedro Henrique S. Figueiredo, Fábio Martins, Vanessa P. Lima, José Sebastião C. Fernandes, Mário Bernardo-Filho, Redha Taiar, Daniel T. Borges, Alessandro Sartorio, Henrique S. Costa, Hércules R. Leite, Vanessa A. Mendonça, Ana Cristina R. Lacerda

**Affiliations:** ^1^Centro Integrado de Pós-Graduação e Pesquisa em Saúde (CIPq-Saúde), Universidade Federal dos Vales do Jequitinhonha e Mucuri (UFVJM), Diamantina, Brazil; ^2^Programa de Pós-Graduação em Reabilitação e Desempenho Funcional (PPGReab), Universidade Federal dos Vales do Jequitinhonha e Mucuri (UFVJM), Diamantina, Brazil; ^3^Programa Multicêntrico de Pós-Graduação em Ciências Fisiológicas (PMPGCF), Universidade Federal dos Vales do Jequitinhonha e Mucuri (UFVJM), Diamantina, Brazil; ^4^Programa de Pós-Graduação em Ciências da Saúde (PPGCS), Universidade Federal dos Vales do Jequitinhonha e Mucuri (UFVJM), Diamantina, Brazil; ^5^Faculdade de Ciências Biológicas e da Saúde, Universidade Federal dos Vales do Jequitinhonha e Mucuri (UFVJM), Diamantina, Brazil; ^6^Faculdade de Ciências Agrárias, Universidade Federal dos Vales do Jequitinhonha e Mucuri (UFVJM), Diamantina, Brazil; ^7^Departamento de Biofísica e Biometria, Universidade do Estado do Rio de Janeiro (UERJ), Rio de Janeiro, Brazil; ^8^Instituto de Biologia Roberto Alcantara Gomes (IBRAG), Rio de Janeiro, Brazil; ^9^GRESPI—EA4694, Reims University, Reims, France; ^10^Departamento de Fisioterapia, Universidade Federal do Rio Grande do Norte (UFRN), Rio Grande do Norte, Brazil; ^11^Division of Auxology and Metabolic Diseases, Istituto Auxologico Italiano, IRCCS (Scientific Institute for Research and Care), Milan, Italy; ^12^Programa de Pós-Graduação em Ciências da Reabilitação, Escola de Educação Física, Fisioterapia e Terapia Ocupacional, Universidade Federal de Minas Gerais (UFMG), Belo Horizonte, Brazil

**Keywords:** whole-body vibration, handgrip strength, surface electromyography, upper limbs, neuromuscular modifications

## Abstract

**Objective:**

To compare the effect of Whole-Body Vibration Exercise (WBVE) applied in push-up modified and half-squat positions, on handgrip strength (HS) and on the electromyography registry (EMGrms) of the flexor digitorum superficialis muscle (FDSM) of the dominant hand.

**Methods:**

Nineteen healthy women (age 23.40 ± 4.03 years, bodyweight: 58.89 ± 9.87 kg), performed in a randomized order five different tests: (S1) Control; (S2) Push-up modified; (S3) Push-up placebo; (S4); Half-squatting; (S5) Half-squatting placebo. The HS and the EMGrms were assessed at baseline and immediately after the tests. ANOVA two-way design mixed test, with Tukey *post hoc*, was used to evaluate the HS, EMGrms and the ratio between EMGrms and HS, i.e., neural ratio (NR). Thus, the lower NR represents the greater neuromuscular modifications. The statistical significance level was set up at *p* < 0.05.

**Results:**

WBVE on S2 increased HS compared to the stimulus applied to the S4 (*p* = 0.0001). The increase in HS was associated with a reduction in the EMGrms of the FDSM (*p* < 0.001) and a lower NR (*p* < 0.0001), i.e., greater neuromuscular modifications, in the S2 compared to the S4 after the tests.

**Conclusion:**

The distance of the stimulus and the positioning on the vibratory platform influence the maximum muscular strength due to neuromuscular modifications of hands in healthy women.

## Introduction

The Whole-body vibration exercise (WBVE) has received plenty of attention for its ability for improving strength and power in the lower-body (Cormie et al., 2006; Avelar et al., 2012; Teles et al., 2015), whereas only a few studies investigated the effects of the vibratory stimulus on upper-body to date (Cochrane and Hawke, 2007; Marín et al., 2010, 2013; Jones et al., 2017). In the clinical and rehabilitation scenario, handgrip strength (HS) can be used for clinical-functional assessment and diagnosis, evolution and progression of the treatment (Shiratori et al., 2014). Furthermore, it can also be utilized as an indicator of overall strength and general health status (Massy-Westropp et al., 2004). In this context, assuming that the WBVE is capable of promoting a strength increase, the study of practices increasing the strength in the upper-body of women becomes extremely important since the higher upper-limbs injuries affect more the females. This is due to the hormonal issues, the double working day, and the lack of muscle preparation for certain tasks (Lowe et al., 2010; Nestler et al., 2017). Furthermore, the hands are important body segments involved with physical performance in different sports (Barut et al., 2008).

Despite the fact that some studies investigated the effect of WBVE on the upper-body musculature (Cochrane and Hawke, 2007; Hazell et al., 2007; Jones et al., 2017; De Souza et al., 2020), a gap in the literature remains regarding transmissibility of the stimulus and the magnitude of its effects. This fact seems to be depended on the position adopted on the vibratory platform. Cochrane and Stannard (2005) suggested that muscles not directly exposed to vibration do not show a concomitant performance enhancement as the vibrated muscles. The vibratory transmission is complex, as vibration signal propagation is influenced by body biomechanics (Kiiski et al., 2008), frequency, amplitude, and postures assumed while on the platform (Rubin et al., 2003). Thus, the effects of body posture regarding neuromuscular modifications on the upper-limb musculature are relatively scarcely studied to date.

Several studies suggested that surface electromyography (EMGrms) might be a useful tool for indirectly evaluate changes of neuromuscular activity during different WBVE stimulus and postures on the vibratory platform (Abercromby et al., 2007a; Di Giminiani et al., 2013, 2014; García-Gutiérrez et al., 2016). To date, most literature regarding EMGrms during WBVE has been focused on the lower extremity. By contrast, few studies have investigated the effects of vibration on the EMGrms of the upper extremity muscles so far (Hand et al., 2009; Di Giminiani et al., 2014; Ashnagar et al., 2016; Morel et al., 2018). Bosco et al. (1999) suggested the use of the ratio between EMGrms and HS, i.e., neural ratio (NR). Thus, the lower NR represents the greater neuromuscular modifications. Although a recent study of our group investigated the effect of WBVE exposure on the efficiency of muscle contraction in the static modified push-up position (De Souza et al., 2020), to our knowledge no study has examined the effect of WBVE exposure on the efficiency of muscle contraction in different postures. These data are relevant in order to understand the possible mechanisms involved in the effects of WBVE on HS. Therefore, the present study aimed to investigate the effect of the WBVE on the static modified push-up position compared to the static half-squat position on HS (primary outcome), EMGrms of the flexor digitorum superficialis muscle (FDSM) of the dominant upper-limb and NR (secondaries outcomes) in healthy women.

## Materials and Methods

### Design

This study was conducted from March to August of 2018. A cross-over study (i.e., all volunteers performed all the five experimental conditions in a randomized order) was performed. To minimize the possibility of bias, opaque envelopes which were sealed with serial-numbers were used. They were opened sequentially and kept in a locked and secure place. The allocation sequence was concealed from the researcher who enrolled and assessed subjects. The design included a familiarization (physical examination, anthropometric measurements, and experience with manual grip strength and procedures performed in subsequent experimental conditions) followed by five tests separated from each other. The periods of recovery were for 24 h over a week to avoid fatigue and bias (Torvinen et al., 2002).

This study was approved by the Human Ethics Committee of the Universidade Federal dos Vales do Jequitinhonha e Mucuri (UFVJM), Diamantina, MG, Brazil (Number = 2,605,760). According to the Helsinki Declaration, International Guidelines for Medical Research Involving Human Beings—CIOMS).

### Subjects

Inclusion criteria were healthy women, sedentary lifestyle, aged between 18 and 40 years. Subjects were considered ineligible if they presented: contraindications to WBVE (i.e., acute inflammations, joint problems, back problems, diabetes, epilepsy, or metabolic or neuromuscular disease) (García-Gutiérrez et al., 2014; Perchthaler et al., 2015) or any discomfort, dizziness, and nausea during the experimental conditions. The individuals were asked to report the use of any medications and were instructed to refrain from participation in strenuous physical activity for 24 h, the consumption of caffeine for 48 h, alcoholic beverages for 24 h, and food for 2 h before tests.

### Intervention

#### Experimental Conditions

**FIGURE 1:**
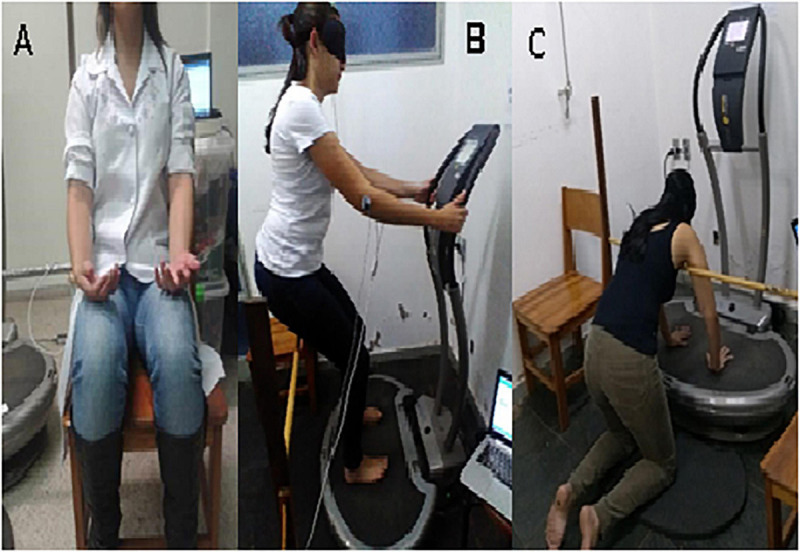
Positioning during the experimental conditions. **(A)** Control, **(B)** Half-squat and Half-squat placebo, **(C)** Push-up modified and Push-up placebo.

The study consisted of five tests: (SI) Control; (S2) Push-up modified (S3) Push-up placebo; (S4) Half-squat; (S5) Half-squat placebo (Figure 1). To minimize the circadian influence, the subjects performed all the five tests at the same time of the day. The trials took place in a thermoneutral environment (22 ± 1°C and 53 ± 2% relative humidity).

(S1) Control. The subjects remained at rest in seating position, feet on the floor and hands in the supine position on the lower limbs, without vibratory stimulus (Figure 1A).

(S2) Push-up modified and (S3) Push-up placebo. In both cases, the subjects postured themselves in the “push-up” position on the vibratory platform with their hands-on platform apart at a distance of 28 cm and 10° elbow flexion (Figure 1B).

(S4) Half-squat and (S5) Half-squat placebo. In these two experimental conditions, the subjects postured themselves in the half-squat position on the vibratory platform with their barefoot apart at a distance of 28 cm, knee flexion of 60° and hands holding side support bar (elbow flexion of 10°) (Figure 1C).

In the S2 and S4 tests, the platform was turned on (vibration parameters: 45 Hz/2 mm/159.73 ms^–2^), and in S3 and S5, the vibratory platform remained disconnected, but with a sonorous stimulus mimicking the vibration.

Each test lasted five continuous minutes with the subjects blindfolded. The WBVE was conducted using a vibratory platform (FitVibe, GymnaUniphy NV, Bilzen, Belgium) with a sinusoidal stimulus. To ensure body positioning and to avoid discharge of weight on the upper limbs, a horizontal bar adjustable according to the subject’s height was used in the push-up and half-squat positions. All vibration parameters (frequency: 45 Hz; amplitude: 2 mm) were selected in agreement with previous studies (Cochrane et al., 2008, 2010). These parameters are reported to have the same values as acceleration during 5 min continuous of vibratory stimulus and raise muscle temperature by 1.5°C, which significantly increases countermovement jump height (9.3%) and power (4.4%).

#### Outcomes Measurements

Before all the tests, each volunteer rested for 15 min with hands in the supine position on the lower-limbs. Thereafter, the subject was allocated to one of the tests.

At baseline and immediately after the tests, the muscle performance and the EMGrms of the FDSM of the dominant hand were analyzed.

##### Handgrip Strength (HS)

**FIGURE 2:**
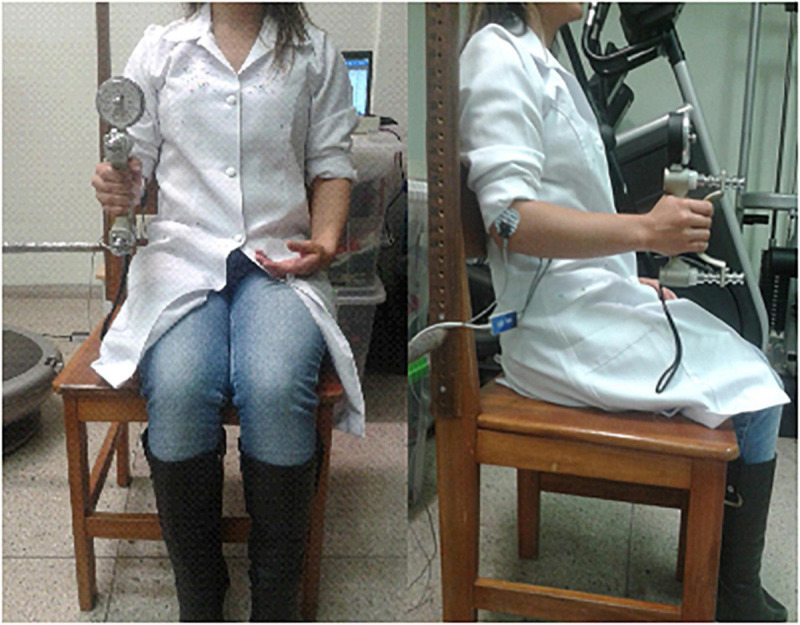
Positioning during the handgrip strength measure with simultaneous electromyography registry of the flexor digitorum superficialis muscle of the dominant hand.

Participants were in a seated position, with the shoulder in adduction and 90° at the elbow joint, forearm in a neutral position. The dominant hand performed three repetitions of 3 s maximum HS (dynamometer, Jamar, United States), a 60 s recovery period being scheduled among the repetitions. HS was determined by the average of the three peak values (Figure 2).

##### Surface Electromyography (EMGs)

For the acquisition of biological signals, we used one channel of the portable EMGs data log instrument (Miotec Equipamentos Biomédicos Ltda, Brazil). The participant preparation consisted of two disposable surface electrodes (Data Hominis Tecnologia Ltda., Brazil) on the muscle belly of the FDSM. In addition, one ground electrode wire attached to the lateral epicondyle of the humerus, with a fixed distance of 20 mm, arranged perpendicular to the direction of muscle fibers. Prior to fixation of the electrodes, the subject’s arms were shaved and the skin was cleaned with 70% alcohol to eliminate residual oil. The muscle was analyzed according to the position described by Surface Electromyography for the Non-Invasive Assessment of Muscles (SENIAM). The analog-to-digital conversion of the signals was performed with a 14-bit input A/D hardware resolution, a sampling frequency of 2 kHz, common rejection module greater than 100 dB, signal-to-noise ratio less than 3 μV and system impedance of 109 Ohms.

Root mean square (RMS) electromyogram (EMGrms) records of the flexor digitorum superficialis muscle of the dominant hand were analyzed at rest and during the HS. The EMG signals were treated filtering with a bandpass filter (4th order Butterworth filter of 10–480 Hz) for signal amplitude analysis, and the data was collected in μV, normalized by peaks (peak-to-peak) of the HS. EMGrms records were transformed in % RMS by software (MiotecSuite 1.0.1065) (Hermens et al., 2000). Both HS and EMGrms were determined concomitantly by the average of the three repetitions performed before and after the experimental conditions. The handgrip strength frequency was of 3,000 ms (i.e., 3 s) with simultaneous and synchronized EMGrms registration. As the EMGrms record pattern takes into account the discard of the first and the last contraction second, we used only the middle contraction second, within the interval range of 1,000–2,000 ms. Thus, all EMGrms record was performed inside 3,000 ms (i.e., time of handgrip strength contraction), but the duration of the signal used to analyze the sliding window of the EMGrms was actually 1,000 ms.

##### Neuronal Ratio (NR)

The NR is the relationship between activation levels of the FDSM (EMGrms) and the HS data obtained from the mean of HS measurement (NR = EMGrms/HS). It represents the electromyogram records (flexor digitorum superficialis muscle electromyography) divided by the mechanical power (HS). The lower is the NR, the greater is the neuromuscular modifications of muscle contraction (Bosco et al., 1999). The ratio of mechanical performance to EMG activity has been used to examine the relative changes in muscle function during repeated-sprint exercise (Racinais et al., 2007; Matsuura et al., 2015). Thus, to investigate the efficiency of muscle contraction, i.e., the ratio between EMGrms of the FDSM and HS (Bosco et al., 1999), is relevant to understand, even partially, the mechanisms related to neuromuscular modifications.

### Statistical Analysis

Intraclass correlation coefficients (ICCs) assessed the test-retest reliability of comparing the mean of the HS between experimental conditions. Shapiro-Wilk’s test for normality and Levene for homogeneity revealed that the data were normally distributed and homogeneous. This experiment with double treatments (baseline and after the WBVE) and five experimental conditions (Control; Push-up modified; Push-up placebo; Half-squat; Half-squat placebo) conducted in only one group (*n* = 19). ANOVA two-way design mixed test, with Tukey *post hoc*, was used to evaluate the primary outcome (HS) and secondary outcomes (EMGrms and NR). The sample size was calculated from a previous study of our group evaluating the dose-response effect of WBV exercise in the push-up position on HS (primary outcome). Thus, a sample size of seventeen participants (mean of difference = 1.42, a standard deviation of difference = 1.93) was required for a power value equal to 80% and a two-tailed α-value = 0.05 for the HS. Nevertheless, once we considered a lost around 15%, the sample size has nineteen participants. The statistical significance level was set at 5%.

## Results

Nineteen healthy women participated in this study (Table 1). All the possible confounding variables were accounted for minimizing bias and variance in the study. The ICC test-retest reliability of HS and electromyographic activity of the FDSM were 0.96 and 0.92, respectively.

**TABLE 1:** Characteristics of the subjects.

**Characteristic**	**(*n* = 19)**
Age (year), mean (SD)	23 (4)
Weight (kg), mean (SD)	58 (9)
Height (m), mean (SD)	1.60 (5)
BMI (kg/m^2^)	22.6 (4.8)

**Data are presented as Mean (SD).**

### Baseline

HS, EMGrms of the flexor digitorum superficialis muscle, and NR were similar at baseline (Table 2).

**TABLE 2:** Effect of whole-body vibration exposure on HS, EMG, and NR.

						***p*-value**				
**Parameters**	**Control**	**Push-up modified**	**Push-up placebo**	**Half-squat**	**Half-squat placebo**	***p1***	***p2***	***p3***	***F***	**η^2^**
HS baseline HS after (N)	26.79 (29.50 − 24.08) 26.23 (28.93 − 23.53)	27.09 (29.69 24.49) 27.91 (30.65 − 25.17)*	28.16 (30.74 − 25.57) 26.18 (28.89 − 23.46)	**27.26 (30.16 − 24.35)**^Δ^ **25.11 (28.06 − 22.16)**	**28.09** (30.75 − 25.43)^Δ^ **25.86** (28.69 − 23.03)	**0.0445**	**0.0001**	**0.0037**	4.80	0.83
EMGrms baseline EMGrms after (% rms)	65.49 (69.76 − 61.22) 64.87 (68.46 − 61.28)	54.42 (61.29 − 47.54) **54.32 (60.94 − 47.70)**	57.80 (61.29 − 47.54) 52.64 (59.39 − 45.89)	64.87 (68.45 − 61.29) 62.35 (65.92 − 58.78)*	69.23 (72.55 − 65.91) 62.43 (65.62 − 59.24)	**<0.001**	0.0500	0.6024	5.35	0.84
NR baseline NR after (% rms.N^–1^)	2.55 (2.84 − 2.26) 2.58 (2.85 − 2.31)	2.08 (2.39 − 1.76) **2.03 (2.35 − 1.71)**	2.06 (2.32 − 1.80) 2.05 (2.32 − 1.77)	2.52 (2.83 − 2.20) 2.68 (3.10 − 2.26)*	2.58 (2.87 − 2.29) 2.56 (2.88 − 2.24)	**<0.0001**	0.7762	0.9107	5.12	0.66

Data are presented as Mean (95% Confidence Interval), i.e., Mean (Upper limit − Lower limit). HS (handgrip strength); EMGrms (electromyography records); NR (neuronal innervation ratio). Measures performed baseline (pre) and after (post) the experimental conditions. Data and p-value in bold indicate statistically significant results. p1, Between-group; p2, Within-group; p3, Interaction. F values. Eta partial η^2^. N = 19 subjects in each experimental coditions. ^Δ^(baseline vs. after): Half-squat and Half-squat placebo. *(after vs. after): Push-up modified and Half-squat.

### Between-Group

WBVE applied in the push-up modified position increased the HS (*p*1 = 0.0445), reduced the EMGrms (*p*1 < 0.001) and reduced the NR (*p*1 < 0.0001) compared to the vibration stimulus applied in the half-squat position (Table 2).

### Within-Group

The HS in both half-squat positions (S4 and S5) was lower after these experimental conditions compared to their respective baseline (*p*2 = 0.0001). There was no within-group effect in EMGrms and NR (Table 2).

### Interaction

There was an interaction between the experimental conditions and treatments only to HS (*p*3 = 0.0037). This was not observed in EMGrms and NR (Table 2).

## Discussion

Studies investigating the effects of WBVE on HS are controversial. To the best of our knowledge, only one study has assessed the effects of WBVE in different positions (30 s of rest and 30 s of WBVE on the push-up, and squat conditions) on HS and muscular activity of FDSM in military soldiers (Morel et al., 2018), and no positive effects being recorded.

Another study, performed in climbers with the aim to investigate the effects of upper-body vibration (26 Hz, 3 mm) on muscular attributes for climbing performance, including HS (Cochrane and Hawke, 2007), did not show a positive effect of upper-body vibration on HS. These authors suggested that climbers might have small margins to improve their performance since their training routine already requires maximum muscular performance. Possibly, it was the same reasoning that the previous study with soldiers did not find any increase of HS in the push-up position. On the other hand, Silva et al. (2017) has investigated the effect of WBVE (single session composed of five series of 30 s vibrations with 1 min rest periods, completed in the position type of modified push up position, with elbows and forearms in a plank position) on the upper-limb performance in complete spinal cord injury (T3 level). The WBVE improved HS comparing pre and post-intervention scores.

The evidence regarding HS indicates that when the vibratory stimulus is applied under the feet, in a half-squat position, the body response is different. In the present study half-squat position, reduced HS and EMGrms after the WBVE, but increased the NR, suggesting that in the half-squat position, the vibratory stimulus that reached the upper limbs was insufficient to promote a more efficient contraction and consequent strength gain. Torvinen et al. (2002) purposed to investigate the effects of WBVE (Biodex System) on muscle function and body balance in healthy subjects. While standing on the platform the subjects repeated four times of the 60 s six differents exercise with feet on the platform. During the investigation of the vibration loading, no statistically significant differences in the HS between the vibration and placebo interventions were found. Thus, the study of Torvinen et al. (2002) and the present study are in line with Cochrane’s and Barut’s studies (Cochrane and Stannard, 2005; Barut et al., 2008), emphasizing the fact that muscles not directly exposed to vibration, does not show a concomitant performance enhancement as the vibrated muscle.

In order to understand how the posture on the platform influences HS, it is relevant to know how the targeted muscle is responding to the vibratory stimulus. Hence the importance of evaluating EMGrms, current studies have suggested that a reduction on EMGrms after vibration stimulus in parallel to the increase in HS can be explained by the neural drive and neuromuscular modifications. Thus, the concept of neuromuscular modifications has been previously used by Milner-Brown et al. (1986) to quantify the excitation-contraction coupling and to determine the level of muscle performance in different populations, i.e., patients with neuromuscular disorders. Moreover, the neuromuscular modifications analysis can objectively evaluate the effects of different therapeutic modalities. The RMS of the EMG signal, which rises with the increased motor unit recruitment and/or firing frequency, is classically considered a global indicator of the neural drive to the active musculature (De Luca, 1997). Moreover, the ratio of mechanical performance to EMG activity has been used previously to examine the relative changes in muscle function during repeated-sprint exercise (Racinais et al., 2007; Matsuura et al., 2015). Thus, the contribution of the NR is relevant to understand, even partially, the mechanisms related to neuromuscular modifications. In this respect, an increase in torque of a muscle contraction can be probably caused by a post-activation potentiation (PAP), i.e., the phenomenon by which muscular performance characteristics are acutely enhanced as the result of their contractile activity prior to a performance test (e.g., handgrip strength). The occurrence of PAP can be explained by the phosphorylation of myosin regulatory ligth chains (CLR) (Baudry and Duchateau, 2007). The possibility that the vibrational stimulus might elicit excitatory inflow through muscle spindle-α-motoneuron connections in the overall motoneuron inflow has also been suggested (Lebedev and Poliakov, 1991). This increase would indicate that this type of treatment is able to stimulate the neuromuscular system more than other treatments used to improve neuromuscular properties (Krol et al., 2011). Our data showed that the increase in HS after vibrational stimulus on push up modified position was accompanied by a reduction in the NR, indicating that the vibratory stimulus promoted neuromuscular modifications.

On the half-squat position, our study presented a lower neuromuscular activation of FDSM, in accordance with the findings of Marín and colleagues (Avelar et al., 2014) who evaluated the effects of performing battle rope exercise with and without the addition of WBVE (Power Plate: 30 and 50 Hz) on muscle activity of the leg, trunk, and upper body in students. The results demonstrated that the EMGrms had a greater increase in the lower-limbs muscles than upper-limbs muscles, and WBVE at 50 Hz increased EMGrms in all muscles measured vs. no WBVE and vs. 30 Hz condition. Thus, the influence of the vibration’s magnitude and of the distance between the focus vibratory to muscle target on the EMGrms were confirmed as factors that seem to influence the results. Moreover, previous studies demonstrated that muscles closer to the vibration inducing system were more receptive to vibration stimulus (Roelants et al., 2006; Nigg, 2010).

Inevitably, this study had some inherent shortcomings. First, the study was performed as a single-center trial with a relatively small number of subjects. However, statistical analyses demonstrated a large effect size and power (Effect size *f* = 2.00, Power = 0.99) for HS (primary outcome), as well as EMGrms (Effect size = 0.83, Power − 0.60) and NR (Effect size *f* = 0.50, Power = 0.24). Moreover, once specific frequency and amplitude were used, the findings of this study cannot be extrapolated to other parameters of vibration as well cannot be generalized to the other group since the volunteers were healthy women. To ensure body positioning and to avoid discharge of weight on the upper limbs, a horizontal bar adjustable according to the subject’s height was used in the push-up and half-squat positions. Thus, the hands were only used in the half-squat and push-up positions to approach with the platform machine minimizing possible muscular fatigue. Nevertheless, the length of the FDSM muscles and the level of contraction during the vibration exposure were factors not controllable in this study.

Based on these results, although WBVE applied in the push-up modified position increased the HS compared to the vibration stimulus applied in the half-squat position; the increase of HS in the push-up modified position was not significant compared to this respective baseline. As a perspective, future studies should measure the muscle temperature or investigate the influence of neural drive using nervous or brain electrical magnetic stimulation in order to understand physiological mechanisms. Nevertheless, the contribution of the neural ratio concomitant with handgrip strength is relevant to understand, even partially, the mechanisms related to neuromuscular modifications. Moreover, we suggest a study with greater sample size or a study about the accumulative effect of several sessions using WBVE (training effect) to conclude that WBVE can be an alternative to improve the HS when applied near the stimulus generated by the platform. However, health professionals should consider the posture in the WBVE aiming at the improvement in HS for healthy women.

## Data Availability Statement

All datasets generated for this study are included in the article/Supplementary Material.

## Ethics Statement

This study was approved by the Human Ethics Committee of the Universidade Federal dos Vales do Jequitinhonha e Mucuri (UFVJM), Diamantina, MG, Brazil (Number = 2,605,760). According to the Helsinki Declaration, International Guidelines for Medical Research Involving Human Beings—CIOMS).

## Author Contributions

LS, VM, AO, FM, and AL conceived and designed the experiments. LS and AO performed the experiments. LS, AO, SF, PF, HL, VM, MB-F, RT, DB, and AL analyzed the data (interpreted results of experiments, prepared figures, drafted manuscript, edited, and revised manuscript) and wrote the manuscript and approved final version of manuscript. AL, HL, and VM contributed materials and analysis tools. All authors contributed to the article and approved the submitted version.

## Conflict of Interest

The authors declare that the research was conducted in the absence of any commercial or financial relationships that could be construed as a potential conflict of interest.
