# Supplementary material for: Whole-Body Vibration Exercise in Different Postures on Handgrip Strength in Healthy Women: A Cross-Over Study
Source: Front Physiol. 2021 Jan 12;11:469499. doi: 10.3389/fphys.2020.469499 (PMC7848817; doi:10.3389/fphys.2020.469499)

Figure. Effect of whole-body vibration exposure on handgrip strength (A), eletromyography records (B), and neuromuscular innervation ratio (C). N= 19 subjects in each experimental condition. Δ (baseline vs after): Half-squat and Half-squat placebo. * (after vs after): Push-up modified and Half-squat.

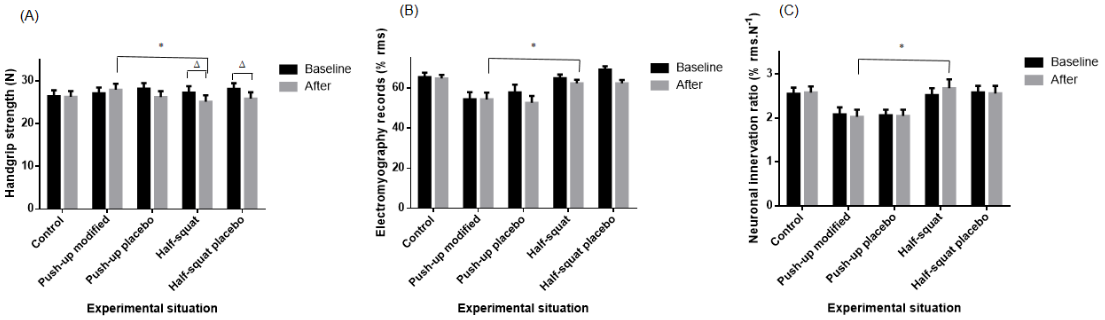

Supplement: Supplementary file 1 [file Data_Sheet_1.PDF]
