# Supplementary material for: Whole-Body Vibration Exercise in Different Postures on Handgrip Strength in Healthy Women: A Cross-Over Study
Source: Front Physiol. 2021 Jan 12;11:469499. doi: 10.3389/fphys.2020.469499 (PMC7848817; doi:10.3389/fphys.2020.469499)
Supplement: Supplementary file 2 [file Table_1.pdf]

HANDGRIP STRENGTH ON BASELINE

Subjects	S1= Control				S2 = Push up modified				S3= Push up placebo				S4 = Half squat				S5= Half squat placebo			
				Mean				Mean				Mean				Mean				Mean
V1	30	30	30	30	28	29	29	29	28	28	26	27	29	27	28	28	28	26	28	27
V2	26	27	27	27	22	26	28	25	30	29	25	28	32	30	30	31	28	28	26	27
V3	32	32	34	33	30	30	32	31	36	36	35	36	30	34	34	33	34	30	34	33
V4	26	25	22	24	22	24	23	23	26	24	24	25	24	22	22	23	22	20	24	22
V5	30	29	28	29	32	32	30	31	28	31	30	30	33	34	33	33	34	34	36	35
V6	19	18	20	19	21	16	16	18	20	18	16	18	17	15	15	16	22	19	20	20
V7	19	16	17	17	21	17	20	19	28	21	26	25	20	16	20	19	20	20	18	19
V8	18	22	22	21	18	22	23	21	19	20	20	20	21	24	20	22	20	20	22	21
V9	30	31	32	31	29	28	29	29	29	31	30	30	30	31	30	30	30	30	32	31
V10	36	38	41	38	35	34	36	35	34	36	36	35	30	30	30	30	33	36	34	34
V11	26	25	26	26	27	28	23	26	27	24	28	26	30	29	26	28	30	31	29	30
V12	23	23	22	23	23	21	24	23	25	28	25	26	27	26	24	26	29	28	24	27
V13	30	30	32	31	40	37	36	38	37	36	37	37	31	33	36	33	35	32	33	33
V14	33	29	22	28	34	26	34	31	36	32	34	34	36	34	34	35	30	30	29	30
V15	20	18	18	19	20	18	20	19	20	19	18	19	22	16	16	18	20	17	17	18
V16	35	35	32	34	35	34	32	34	34	35	34	34	38	36	36	37	34	36	34	35
V17	22	20	22	21	26	24	20	23	26	27	26	26	24	23	20	22	22	24	22	23
V18	27	18	27	24	22	22	38	27	23	26	26	25	22	20	20	21	33	34	36	34
V19	35	35	35	35	32	36	30	33	36	32	34	34	34	34	36	35	34	35	35	35

HANDGRIP STRENGTH AFTER WBVE																				
	S1= Control				S2 = Push up modified				S3= Push up placebo				S4 = Half squat				S5= Half squat placebo			
Subject				Mean				Mean				Mean				Mean				Mean
V1	29	26	31	29	28	26	27	27	27	26	26	26	24	26	29	26	28	26	27	27
V2	28	27	26	27	30	29	28	29	28	26	29	28	26	28	28	27	22	25	26	24
V3	34	34	32	33	32	31	34	32	37	34	31	34	31	29	30	30	30	32	32	31
V4	24	22	20	22	24	24	23	24	22	21	22	22	23	20	20	21	24	21	20	22
V5	27	28	29	28	32	30	32	31	31	30	22	28	33	32	31	32	32	33	32	32
V6	22	18	16	19	18	19	22	20	16	16	16	16	18	15	16	16	19	14	20	18
V7	17	16	20	18	20	18	19	19	20	20	22	21	17	15	16	16	15	19	20	18
V8	18	22	20	20	24	21	23	23	18	20	20	19	18	20	18	19	20	20	19	20
V9	28	30	28	29	30	30	33	31	28	28	28	28	28	30	32	30	25	28	26	26
V10	36	36	36	36	36	37	36	36	34	35	36	35	24	30	34	29	28	33	34	32
V11	29	30	27	29	28	28	27	28	22	29	24	25	25	28	27	27	26	24	26	25
V12	18	22	19	20	20	22	22	21	24	22	24	23	21	24	22	22	21	22	26	23
V13	30	32	34	32	36	38	36	37	32	30	33	32	30	37	35	34	35	36	36	36
V14	28	26	28	27	38	33	34	35	34	33	32	33	28	32	28	29	28	28	24	27
V15	20	18	18	19	22	18	21	20	20	17	20	19	14	13	14	14	15	16	14	15
V16	34	32	34	33	32	37	35	35	34	34	36	35	35	35	29	33	33	35	32	33
V17	22	22	23	22	26	28	26	27	20	24	25	23	21	18	18	19	20	17	19	19
V18	20	22	23	22	22	20	22	21	15	26	16	19	19	20	18	19	31	34	22	29
V19	34	34	36	35	34	34	36	35	31	32	34	32	33	34	32	33	34	36	34	35

EMGrms during HANDGRIP STRENGTH - BASELINE																				
Subjects	S1= control				S2 = Push-up modified				S3 = Push-up placebo				S4 = Half-squat				S5 = Half-squat placebo			
				Mean				Mean				Mean				Mean				Mean
V1	42.28	69.50	68.32	60.03	75.62	66.80	68.74	70.39	37.47	35.13	36.07	36.22	76.62	65.28	65.24	69.05	71.97	70.49	73.48	71.98
V2	82.60	69.93	75.58	76.04	60.07	59.54	62.12	60.58	37.75	34.94	33.04	35.24	71.24	63.97	61.97	65.73	76.99	67.56	74.32	72.96
V3	71.15	61.71	61.79	64.88	84.84	68.41	66.47	73.24	74.59	59.69	53.17	62.48	54.48	58.37	51.88	54.91	74.37	75.09	78.47	75.98
V4	42.82	36.33	31.88	37.01	68.10	56.53	55.04	59.89	72.71	69.62	57.91	66.75	76.74	65.55	65.09	69.13	74.32	70.42	75.20	73.31
V5	75.67	67.00	72.97	71.88	44.35	39.93	36.02	40.10	60.06	73.65	70.05	67.92	68.47	68.97	57.61	65.02	70.35	75.91	72.41	72.89
V6	61.54	62.91	59.40	61.28	31.30	29.45	29.94	30.23	36.95	21.74	28.03	28.91	71.94	66.16	45.95	61.35	56.63	55.13	56.20	55.99
V7	77.12	73.90	75.61	75.54	72.45	53.87	73.98	66.77	72.01	59.42	70.47	67.30	58.05	51.90	59.61	56.52	61.63	76.59	62.64	66.95
V8	61.98	71.60	61.34	64.97	58.48	60.30	73.95	64.24	55.57	77.97	66.28	66.61	52.95	52.38	46.47	50.60	67.17	73.66	67.73	69.52
V9	74.78	72.61	71.71	73.03	62.01	64.73	63.79	63.51	85.40	73.86	70.28	76.51	67.48	71.09	58.61	65.73	76.77	58.96	64.92	66.88
V10	79.74	79.46	79.30	79.50	34.35	32.81	32.89	33.35	66.49	75.26	66.37	69.37	68.34	65.43	62.15	65.31	65.11	74.68	62.89	67.56
V11	64.57	60.84	57.78	61.06	38.53	33.57	33.49	35.20	48.34	47.28	48.73	48.12	61.85	66.91	60.10	62.95	68.03	69.08	69.33	68.81
V12	71.26	65.59	63.93	66.93	29.22	26.77	27.02	27.67	61.72	71.52	56.25	63.16	72.56	73.92	76.98	74.49	71.09	52.27	49.25	57.54
V13	66.44	54.94	65.04	62.14	55.71	53.69	54.29	54.56	80.86	69.52	64.13	71.50	55.70	55.92	55.78	55.80	57.99	53.14	51.95	54.36
V14	77.48	64.96	52.63	65.02	70.34	49.97	57.39	59.23	83.52	58.9	64.77	69.06	76.07	82.32	64.04	74.14	83.51	68.78	61.81	71.37
V15	66.39	52.28	55.35	58.01	58.42	57.75	58.5	58.22	23.54	23.45	22.65	23.21	82.96	65.86	75.32	74.71	67.43	73.41	62.34	67.73
V16	82.05	64.88	58.44	68.46	86.28	79.98	78.91	81.72	79.76	67.91	61.02	69.56	92.53	79.37	76.15	82.68	83.02	82.06	75.86	80.31
V17	56.07	59.38	57.21	57.55	45.87	45.5	48.58	46.65	38.6	37.3	36.49	37.46	73.41	54.78	57.41	61.87	86.33	76.46	86.3	83.03
V18	62.55	49.42	85.22	65.73	54.64	50.89	70.00	58.51	61.86	58.09	71.91	63.95	75.45	78.47	36.00	63.31	85.75	60.41	52.76	66.31
V19	74.82	69.31	81.55	75.23	54.8	42.91	52.19	49.97	88.73	62.99	72.67	74.80	57.56	60.01	60.39	59.32	71.70	78.63	65.23	71.85

EMGrms during HANDGRIP STRENGTH - AFTER WBVE																				
	**S1= control**				**S2 = Push-up modified**				**S3 = Push-up placebo**				**S4 = Half-squat**				**S5 = Half-squat placebo**			
Subjects				Mean				Mean				Mean				Mean				Mean
V1	68.10	67.90	74.09	70.03	67.14	62.69	54.67	61.50	36.55	37.29	38.99	37.61	60.42	62.35	40.61	54.46	62.18	65.03	62.63	63.28
V2	76.80	71.10	73.49	73.80	60.97	59.84	60.15	60.32	34.09	33.41	33.88	33.79	72.82	61.50	59.76	64.69	66.02	69.45	71.58	69.02
V3	72.78	61.86	61.62	65.42	70.41	68.10	74.05	70.85	64.58	56.07	53.42	58.02	80.27	69.62	79.03	76.31	64.75	63.12	54.63	60.83
V4	54.32	44.28	38.72	45.77	68.61	53.20	56.87	59.56	57.05	46.75	47.51	50.44	54.64	49.22	64.73	56.20	74.82	55.95	59.03	63.27
V5	68.58	69.73	70.92	69.74	34.51	30.73	37.86	34.37	76.35	67.24	53.54	65.71	58.76	59.32	62.36	60.15	66.30	69.17	76.20	70.56
V6	63.66	48.27	43.05	51.66	23.27	24.40	23.17	23.61	23.59	18.00	25.14	22.24	56.75	53.24	69.09	59.69	57.15	53.03	65.87	58.68
V7	78.11	71.72	70.05	73.29	67.42	59.86	62.78	63.35	56.50	57.57	65.50	59.86	57.03	49.70	60.11	55.61	64.25	66.10	70.37	66.91
V8	68.46	56.54	66.65	63.88	66.34	66.98	77.79	70.37	49.92	64.81	70.34	61.69	45.37	41.92	41.93	43.07	55.92	70.51	59.47	61.97
V9	69.07	64.29	70.07	67.81	78.05	64.86	70.81	71.24	67.69	67.57	68.22	67.83	63.84	70.68	68.93	67.82	42.91	44.14	47.85	44.97
V10	75.20	64.54	74.18	71.31	37.53	31.29	30.39	33.07	56.47	54.66	59.69	56.94	57.17	67.08	68.92	64.39	65.08	65.90	64.27	65.08
V11	69.08	75.47	62.66	69.07	30.62	30.02	39.42	33.35	37.90	48.13	47.54	44.52	52.55	71.63	64.25	62.81	55.26	53.58	56.38	55.07
V12	61.90	69.97	59.51	63.79	25.25	28.40	75.47	43.04	56.57	52.04	50.19	52.93	64.67	66.72	63.18	64.86	44.03	58.45	56.24	52.91
V13	62.31	62.91	69.24	64.82	54.30	54.87	54.94	54.70	69.30	66.16	66.61	67.36	54.50	56.93	56.05	55.83	52.98	64.40	69.46	62.28
V14	66.07	59.44	58.53	61.35	58.23	52.33	43.69	51.42	69.21	64.58	57.15	63.65	62.68	61.11	60.14	61.31	71.96	60.45	63.27	65.23
V15	67.75	58.73	58.48	61.65	59.90	59.45	59.73	59.69	26.40	21.54	26.45	24.80	65.72	76.54	77.51	73.26	44.87	63.69	53.88	54.15
V16	56.66	51.62	55.91	54.73	72.20	68.49	78.30	73.00	73.06	53.87	66.36	64.43	67.15	68.68	63.76	66.53	62.64	73.77	59.63	65.35
V17	61.59	56.94	65.56	61.36	46.85	43.90	49.18	46.64	34.74	37.32	36.96	36.34	65.28	72.23	63.6	67.04	77.06	75.47	77.18	76.57
V18	69.69	60.17	57.6	62.49	62.53	58.17	62.90	61.20	59.88	78.25	48.44	62.19	76.27	70.85	74.38	73.83	73.04	66.46	51.37	63.62
V19	79.94	75.77	80.78	78.83	60.69	64.16	57.52	60.79	69.44	70.65	69.11	69.73	57.39	58.64	54.61	56.88	71.33	68.50	59.74	66.52

Neuronal innervation ratio = EMGrms / HS										
	**Baseline**					**After WBVE**				
Subjects	**Control**	**Push up Placebo**	**Half-squat placebo**	**Push-up modified**	**Half-squat**	**Control**	**Push up Placebo**	**Half-squat placebo**	**Push-up modified**	**Half-squat**
V1	2.00	1.33	2.63	2.46	2.47	2.44	1.43	2.34	2.28	2.07
V2	2.85	1.26	2.67	2.39	2.14	2.73	1.22	2.84	2.08	2.37
V3	1.99	1.75	2.33	2.39	1.68	1.96	1.71	1.94	2.19	2.54
V4	1.52	2.71	3.33	2.60	3.05	2.08	2.33	2.92	2.52	2.68
V5	2.48	2.29	2.10	1.28	1.95	2.49	2.38	2.18	1.10	1.88
V6	3.23	1.61	2.75	1.71	3.92	2.77	1.39	3.32	1.20	3.65
V7	4.36	2.69	3.46	3.45	3.03	4.15	2.90	3.72	3.33	3.48
V8	3.14	3.39	3.36	3.06	2.34	3.19	3.19	3.15	3.10	2.31
V9	2.36	2.55	2.18	2.22	2.17	2.37	2.42	1.71	2.30	2.26
V10	2.07	1.96	1.97	0.95	2.18	1.98	1.63	2.06	0.91	2.20
V11	2.38	1.83	2.29	1.35	2.22	2.41	1.78	2.17	1.21	2.36
V12	2.95	2.43	2.13	1.22	2.90	3.24	2.27	2.30	2.02	2.90
V13	2.03	1.95	1.63	1.45	1.67	2.03	2.13	1.75	1.49	1.64
V14	2.32	2.03	2.41	1.89	2.14	2.24	1.93	2.45	1.47	2.09
V15	3.11	1.22	3.76	3.01	4.15	3.30	1.31	3.61	2.94	5.36
V16	2.01	2.03	2.32	2.43	2.26	1.64	1.86	1.96	2.11	2.02
V17	2.70	1.42	3.66	2.00	2.77	2.75	1.58	4.10	1.75	3.53
V18	2.74	2.56	1.93	2.14	3.06	2.88	3.27	2.19	2.87	3.89
V19	2.15	2.20	2.07	1.53	1.71	2.27	2.16	1.92	1.75	1.72
